# Right Ventricular Septal Pacing: Has it come of age?

**Published:** 2010-02-01

**Authors:** Johnson Francis, B Jayesh, M Ashishkumar, Ali Faizal, Harry Mond

**Affiliations:** 1 Malabar Institute of Medical Sciences, Calicut, Kerala, India; 2 The Royal Melbourne Hospital, Parkville, Victoria, Australia

**Keywords:** right ventricular septal pacing, alternate site pacing

Prolonged pacing from the right ventricular (RV) apex has been shown to be associated with progressive left ventricular dysfunction as demonstrated by heart failure, atrial fibrillation and an increased morbidity and mortality [1-6].  This has led to an interest in alternate RV pacing sites and in particular the mid RV septum and the RV outflow tract (RVOT) septum [7-11]. These sites are theoretically associated with a more physiological ventricular activation. Despite the perceived advantages of septal pacing, results to date are not confirmatory [12-18]. These studies were generally acute or extended to 6-months and the leads secured to the RVOT and thus were not necessarily septal. On review of the early work of Durrer et al in 1970 [19] the septal regions of the RVOT and mid RV are the first zones of the ventricle to depolarize, suggesting that pacing from these areas on the right side of the septum would achieve as normal a contraction pattern as possible. In contrast, the free wall of the RV is the last zone to be depolarized. When attempting to prove the physiologic and hemodynamic benefits of septal pacing, it seems illogical to choose the RVOT with a mix of both septal and free wall pacing. The potential benefits of septal pacing would possibly be negated by free wall pacing and thus it is not surprising that there has been no consistent benefit over RV apical pacing demonstrated.

These early reported studies involved using a lead stylet shaped with a simple curve which has been shown to position only 61% of leads onto the RV septum with the remainder on the anterior or free walls [8]. Hence we need to design a tool to consistently place the leads onto the RV septum.  On review of the anatomy of the RV, the septum lies posteriorly with the free wall in front and separating them is the anterior wall, where the left anterior descending coronary artery lies [9,10,20].  A pacing lead being positioned on the RV septum must therefore pass backwards after traversing the tricuspid valve. Such a stylet directing a pacing lead in this fashion has now been designed and commercially available (Model 4140, St Jude Medical, Sylmar, California) [11]. The RV zones where long term pacing has been successful include the lower portion of the RVOT septum or the mid septum [11]. Clinical results show an extremely low incidence of lead dislodgement [9], excellent long term stimulation thresholds [8] and no perforation, pericarditis or pericardial tamponade. Of particular importance is the recognition of RV septal positioning using the fluoroscopic 40º left anterior oblique view during implantation [9,21].

Now that we have the surgical tools for lead placement onto the RV septum and a simple method of confirmation, it behooves us as physicians to repeat the earlier studies, but this time confirming septal positioning. One concern, however, is how long the clinical trials should be conducted?  As stated earlier, most studies were either acute or lasted about six months. Tse et al  [22] compared pacing from the RVOT with RV apex and differences were not significant until 18-months post implant. Lewicka-Nowak et al [23] conducted a small 7-year follow up of 27 patients randomized between RVOT pacing and RV apical pacing. Although once again the cases were not necessarily septal, there was a significant drop in left ventricular ejection fraction with RV apical pacing whilst no drop was noted with RVOT pacing. The NT-pro BNP levels were also significantly higher and there was more tricuspid regurgitation in the RV apical pacing group. These studies suggest that future studies should be conducted for a minimum of two years.

To determine the optimal site for RV pacing, two multicenter randomized trials are currently underway. These are the Right Ventricular Apical and High Septal Pacing to Preserve Left Ventricular Function (Protect Pace), and Right Ventricular Apical versus Septal Pacing (RASP) trials. In Protect Pace, enrollment is almost complete and the mid septum is the pacing site. The RASP study has the inflow septum as the pacing site, The two studies have different study designs and protocols but all will analyze the long term (24 - 36 months) effects of RV pacing on LV performance indices and functional capacity, with changes in LVEF being the primary outcome [24]. Let us hope that these trials will shed more light on the benefits of RV septal pacing. A third trial; the Optimize RV Selective Site Pacing Clinical Trial (Optimize RV) has recently been abandoned.

## References

[R1] Nielsen JC (2003). A randomized comparison of atrial and dual-chamber pacing in 177 consecutive patients with sick sinus syndrome: echocardiographic and clinical outcome. J Am Coll Cardiol.

[R2] Wilkoff  BL (2002). Dual-chamber pacing or ventricular backup pacing in patients with an implantable defibrillator: the Dual Chamber and VVI Implantable Defibrillator (DAVID) Trial. JAMA.

[R3] Udink ten Cate FE (2001). Dilated cardiomyopathy in isolated congenital complete atrioventricular block: early and long-term risk in children. J Am Coll Cardiol.

[R4] Moak JP (2001). Congenital heart block: development of late-onset cardiomyopathy, a previously underappreciated sequela. J Am Coll Cardiol.

[R5] Sweeney MO (2003). Adverse effect of ventricular pacing on heart failure and atrial fibrillation among patients with normal baseline QRS duration in a clinical trial of pacemaker therapy for sinus node dysfunction. Circulation.

[R6] Andersen HR (1997). Long-term follow-up of patients from a randomized trial of atrial versus ventricular pacing for sick-sinus syndrome. Lancet.

[R7] Lieberman R (2004). Selective Site Pacing: Defining and Reaching the Selected Site. Pacing Clin Electrophysiol.

[R8] McGavigan AD (2006). Right Ventricular Outflow Tract Pacing: Radiographic and Electrocardiographic Correlates of Lead Position. Pacing Clin Electrophysiol.

[R9] Mond HG (2007). The Right Ventricular Outflow Tract: The Road to Septal Pacing. Pacing Clin Electrophysiol.

[R10] Hillock RJ (2007). The Right Ventricular Outflow Tract: A Comparative Study of Septal, Anterior Wall and Free Wall Pacing. Pacing Clin Electrophysiol.

[R11] Rosso R (2010). Right Ventricular Septal Pacing: The Success of Stylet-Driven Active- Fixation Leads. Pacing Clin Electrophysiol.

[R12] Karpawich PP (1997). Comparative left ventricular function following atrial, septal, and apical single chamber heart pacing in the young. Pacing Clin Electrophysiol.

[R13] Schwaab B (1999). Influence of right ventricular stimulation site on left ventricular function in atrial synchronous ventricular pacing. J Am Coll Cardiol.

[R14] Mera F (1999). A comparison of ventricular function during high right ventricular septal and apical pacing after his-bundle ablation for refractory atrial   fibrillation. Pacing Clin Electrophysiol.

[R15] Victor F (2006). A randomized comparison of permanent septal versus apical right ventricular pacing: short-term results. J Cardiovasc Electrophysiol.

[R16] Stambler BS (2003). Right ventricular outflow versus apical pacing in pacemaker patients with congestive heart failure and atrial fibrillation. J Cardiovasc Electrophysiol.

[R17] Gong X (2009). Is Right Ventricular Outflow Tract Pacing Superior to Right Ventricular Apex Pacing in Patients with Normal Cardiac Function?. Clin Cardiol.

[R18] Yoon HJ (2009). Acute changes in cardiac synchrony and output according to RV pacing sites in Koreans with normal cardiac function. Echocardiography.

[R19] Durrer D (1970). Total Excitation of the Isolated Human Heart. Circulation.

[R20] Teh AW (2009). Pacing from the Right Ventricular Septum: Is There a Danger to the Coronary Arteries?. Pacing Clin Electrophysiol.

[R21] Medi C (2009). Right Ventricular Outflow Tract Pacing: Long-Term Follow-Up of Ventricular Lead Performance. Pacing Clin Electrophysiol.

[R22] Tse HF (1997). Long Term Effect of Right Ventricular Pacing on Myocardial Perfusion and Function. J Am Coll Cardiol.

[R23] Lewicka-Nowak E (2006). Right ventricular apex versus right ventricular outflow tract pacing: prospective, randomised, long-term clinical and echocardiographic evaluation. Kardiol Pol.

[R24] Kaye G (2009). Search for the optimal right ventricular pacing site: design and implementation of three randomized multicenter clinical trials. Pacing Clin Electrophysiol.

